# Correlation of Membrane Binding and Hydrophobicity to the Chaperone-Like Activity of PDC-109, the Major Protein of Bovine Seminal Plasma

**DOI:** 10.1371/journal.pone.0017330

**Published:** 2011-03-08

**Authors:** Rajeshwer S. Sankhala, Rajani S. Damai, Musti J. Swamy

**Affiliations:** School of Chemistry, University of Hyderabad, Hyderabad, Andhra Pradesh, India; University of South Florida College of Medicine, United States of America

## Abstract

The major protein of bovine seminal plasma, PDC-109 binds to choline phospholipids present on the sperm plasma membrane upon ejaculation and plays a crucial role in the subsequent events leading to fertilization. PDC-109 also shares significant similarities with small heat shock proteins and exhibits chaperone-like activity (CLA). Although the polydisperse nature of this protein has been shown to be important for its CLA, knowledge of other factors responsible for such an activity is scarce. Since surface exposure of hydrophobic residues is known to be an important factor which modulates the CLA of chaperone proteins, in the present study we have probed the surface hydrophobicity of PDC-109 using bisANS and ANS. Further, effect of phospholipids on the structure and chaperone-like activity of PDC-109 was studied. Presence of DMPC was found to increase the CLA of PDC-109 significantly, which could be due to the considerable exposure of hydrophobic regions on the lipid-protein recombinants, which can interact productively with the nonnative structures of target proteins, resulting in their protection. However, inclusion of DMPG instead of DMPC did not significantly alter the CLA of PDC-109, which could be due to the lower specificity of PDC-109 for DMPG as compared to DMPC. Cholesterol incorporation into DMPC membranes led to a decrease in the CLA of PDC-109-lipid recombinants, which could be attributed to reduced accessibility of hydrophobic surfaces to the substrate protein(s). These results underscore the relevance of phospholipid binding and hydrophobicity to the chaperone-like activity of PDC-109.

## Introduction

In mammals, the secretions of male accessory glands, testes and epididymis – collectively termed as seminal plasma – serve to transport spermatozoa from the male testes to the female uterus, which leads to fertilization. Seminal plasma is made up of organic as well as inorganic molecules of low and high molecular weight; while the low molecular weight fraction contains a wide variety of chemical species, proteins are the only high molecular weight constituents present in it [1]. Freshly ejaculated spermatozoa are unable to fertilize the egg. They acquire fertilizing competence when they undergo ‘capacitation’, which occurs during their transit through the female genital tract [2]. The mammalian seminal plasma contains various proteinaceous decapacitation factors which prevent inappropriate acrosomal reactions [3] and inactivation of such factors might modulate the sperm capacitation *in vivo*, thereby enhancing the fertilizing capacity of spermatozoa.

Among the various mammalian species, extensive studies have been carried out on the proteins from bovine seminal plasma. The bovine seminal plasma contains four acidic proteins designated as BSP-A1, BSP-A2, BSP-A3 and BSP-30 kDa, which are collectively referred to as *bovine seminal plasma proteins* or *BSP proteins* [4]–[6]. All BSP proteins have an *O*-glycosylated *N*-terminus of variable length, except BSP-A3 which is not glycosylated [5]. BSP-A1 and BSP-A2 have identical primary structure and differ only in the degree of glycosylation, and a mixture of these two proteins is also referred to as PDC-109 [7], [8]. The BSP proteins bind with high specificity to choline-containing phospholipids such as phosphatidylcholine and sphingomyelin present on the sperm plasma membrane [9]. This binding leads to the extraction of choline phospholipids and cholesterol from sperm plasma membrane, a process referred to as *cholesterol efflux*, which is an important step in sperm capacitation [10], [11].

PDC-109 is a protein with 109 amino acid residues and is made up of two tandemly repeating fibronectin type-II (FnII)^1^ domains of 38–41 amino acids, each containing two disulfide bridges and one phosphorylcholine binding site [7], [12], [13]. Single-crystal X-ray diffraction studies demonstrated that both the choline-binding sites are present on the same face of the protein [14]. Binding of PDC-109 to phospholipid membranes and soluble ligands has been characterized extensively by biophysical approaches [15]–[21]. PDC-109 appears to be a multifunctional protein as it has been reported to bind a variety of other ligands including apolipoproteins A1 and A2, heparin, several types of collagens etc [4], [6], [22].

Very recently we have demonstrated that PDC-109 can function as a molecular chaperone [23]. The term ‘Molecular Chaperone’ was originally coined for proteins which prevent the aggregation of other proteins under conditions of stress and restore their functional activity on removal of stress [24]–[31]. These proteins assist the non-covalent folding/unfolding and the assembly/disassembly of other proteins, but are not associated with the latter when they perform their normal biological functions [32]–[34]. An important class of molecular chaperones are small heat shock proteins (sHSP) which exist as large multimeric structures of 300–1000 kDa in size, with subunits of 12–43 kDa [35], [36]. Most general features of molecular chaperones include polydispersity, significant surface hydrophobicity and presence of unordered structural components [35], [37], [38]. PDC-109 is also a polydisperse protein and forms oligomeric structures as large as 150 kDa [39]. Polydispersity appears to be essential for the chaperone-like activity (CLA) of PDC-109, since dissociation of such large oligomers into smaller ones results in the loss of CLA [23]. X-ray diffraction studies showed that PDC-109 possesses a significant amount of unordered structure, whereas CD and FTIR studies indicate that this protein undergoes conformational changes upon membrane binding [14], [15], [39]. Such conformational flexibility may be relevant to the CLA of PDC-109 wherein it interacts with target proteins to protect them against various types of stress [23].

Although our earlier studies clearly demonstrated that PDC-109 exhibits chaperone-like activity *in vitro* [23], how this protein facilitates the proper folding of the target proteins or prevents their misfolding/aggregation is not clear. Several studies suggest the involvement of surface exposed hydrophobic residues on various chaperones, in their association with partially unfolded proteins [32]–[34]. The exposure of hydrophobic regions on the surface of α-crystallin has been attributed to be responsible for enhanced CLA, suggesting the role of hydrophobicity in the chaperone like function of sHSP [32]–[34]. 4,4′-Dianilino-1,1′-binaphthyl-5,5′-disulphonic acid (bisANS) and 8-anilinonaphthalene sulphonic acid (ANS) have been widely used to measure the surface hydrophobicity of various proteins. Binding of PDC-109 and its domain B to phospholipid membranes and partial insertion of sections of the protein, including a segment containing Trp93 into the hydrophobic interior of membranes indicated the presence of hydrophobic stretches in this protein [16], [17], [40], [41]. In view of this, in the present study we have investigated the binding of bisANS and ANS with PDC-109 using isothermal titration calorimetry and steady state fluorescence spectroscopy, respectively. Further, the effect ANS binding on the conformation and CLA of PDC-109 was also investigated. Since PDC-109 binds to phospholipid membranes, it is of interest to study the possible role of such interactions on the CLA, which was investigated with a number of target proteins such as alcohol dehydrogenase (ADH), carbonic anhydrase (CA) and lactate dehydrogenase (LDH). Presence of dimyristoylphosphatidylcholine (DMPC) – which is specifically recognized by PDC-109 – resulted in a multifold increase in the CLA of the protein. ANS and bisANS binding to DMPC-PDC-109 mixture revealed enhanced exposure of hydrophobic surfaces, which could be responsible for such a dramatic change in the CLA. Cholesterol which makes lipid membranes in the liquid-crystalline phase more rigid was found to decrease the CLA of PDC-109/DMPC recombinants. Overall, our present results provide a detailed characterization of the hydrophobicity of PDC-109 and demonstrate that phospholipid binding nature of PDC-109 could also be relevant to its chaperone-like activity, *in vivo*.

## Materials and Methods

### Materials

ANS, bisANS, aldolase, carbonic anhydrase, and DEAE Sephadex A-25 were from Sigma (St. Louis, MO). Sephadex G-50 (superfine) was obtained from Pharmacia (Uppsala, Sweden). DMPC, DMPG and cholesterol were purchased from Avanti Polar Lipids (Alabster, AL). LDH, ADH, tris base and other chemicals were purchased from local suppliers and were of the highest purity available. PDC-109 was purified by a combination of gel filtration on Sephadex G-50 and affinity chromatography on DEAE Sephadex A-25 as described earlier [16], [42]. The purified protein was dialyzed extensively against 50 mM Tris buffer, 0.15 M NaCl, 5 mM EDTA, pH 7.4 (TBS-I) and stored at 4°C. All experiments were performed in TBS-I unless stated otherwise.

### Inhibition of thermal aggregation of aldolase by PDC-109

The chaperone-like activity of PDC-109 was assayed by monitoring its ability to prevent heat-induced aggregation of aldolase at 48°C [23], [27]. Aggregation of aldolase was monitored by recording light scattering at 360 nm as a function of time using a PerkinElmer Lambda 35 UV/Vis spectrophotometer, which was also used for all other aggregation assays reported in this study as well as for all the absorption measurements. Aldolase (0.93 µM) was pre-incubated for 5 minutes in the presence and absence of PDC-109 at room temperature before the assay. Three different samples with PDC-109:aldolase molar ratios of 1∶2.4, 1∶1.2 and 1∶0.6 were used. Aggregation profile for the native enzyme was taken as 100% and percent aggregation of other samples was calculated with respect to that of the native enzyme.

### Isothermal titration calorimetric study of bisANS binding to PDC-109

The binding of bisANS to PDC-109 was studied at 30°C by isothermal titration calorimetry (ITC) using a Microcal VP-ITC instrument (MicroCal LLC, Northampton, MA, USA). In a typical experiment, 25 aliquots (5 µL each) of 5 mM bisANS were injected consecutively from the syringe into the calorimeter cell containing 25 µM PDC-109. To minimize the contribution from heat of dilution to the measured binding enthalpy, the ligand and protein solutions were prepared in the same buffer. Injections were made at intervals of 300 sec, and to ensure proper mixing after each injection, a constant stirring speed of 300 rpm was maintained during the experiment. Control experiments were performed by titrating bisANS into the buffer solution and the resulting heat changes were subtracted from measured heats of binding. Solutions were degassed under vacuum prior to their use in the calorimetric titrations. The titration data were analyzed by using the *sequential binding model* available in the Origin software provided by MicroCal, which yielded the following parameters associated with the bisANS/PDC-109 interaction: equilibrium association constant (*K*_a_), enthalpy (Δ*H*), entropy (Δ*S*) and the stoichiometry of binding (*n*, which corresponds to the number of bisANS molecules bound per molecule of PDC-109).

### Fluorescence studies on ANS binding to PDC-109

Binding of ANS to PDC-109 was investigated by fluorescence titrations monitoring the fluorescence intensity of the probe. Measurements were performed at 25°C using a Spex Fluoromax-4 spectrofluorimeter with excitation and emission slits of 2 and 10 nm, respectively. Titrations were performed by adding small aliquots of ANS from a 1 mM stock solution to 2.0 mL of PDC-109 (OD_280_≅0.05) in the cuvette. Samples were excited at 350 nm and emission spectra were recorded between 400 and 600 nm. All the binding data reported here correspond to the average values obtained from two independent titrations. Fluorescence intensities were corrected for dilution effects and for fluorescence of free ligand determined in parallel titrations without the protein.

### Effect of ANS binding on the conformation and chaperone-like activity of PDC-109

A 6.0 mg/ml solution of PDC-109 was incubated with 10 mM ANS for 30 minutes at ambient temperature. The solution was passed through a Sephadex G-50 column [(10×0.5) cm] to remove unbound ANS. Protein concentration in the eluted fractions was determined by Lowry assay [43], whereas the concentration of bound ANS was estimated using its molar absorption coefficient of 5×10^3^ M^−1^.cm^−1^ at 350 nm. The effect of ANS binding on the secondary structures of PDC-109 was analyzed by CD spectroscopy using a Jasco J-810 spectropolarimeter fitted with a thermostatted cell holder and interfaced to a thermostatic waterbath. Far UV CD spectra were recorded at 25°C using a protein concentration of 0.12 mg/mL at a scan rate of 50 nm/min using a 2 mm pathlength quartz cell and a slit width of 2 nm. All spectra were corrected with appropriate buffer blanks and data are expressed as mean residual ellipticities.

Effect of ANS binding on the CLA of PDC-109 was investigated by LDH aggregation assay [23]. In a typical experiment 0.15 mg/mL of LDH was mixed with a fixed concentration (0.05 or 0.075 mg/mL) of native PDC-109 and allowed to stand for 5 minutes at room temperature, followed by incubation at 48°C. Heat induced aggregation of the enzyme was assessed by monitoring the increase in sample turbidity at 360 nm as a function of time. Experiments with ANS bound PDC-109 were performed at similar concentrations as mentioned above and then compared with the activity of the native protein. Effect of ANS alone on the aggregation of the target protein was used as a positive control. Aggregation of native LDH was taken as 100% and the aggregation of other samples was normalized with respect to it.

### Phospholipid induced modulation of the CLA and tertiary structure of PDC-109

Unilamellar vesicles of DMPC and DMPG were used in studies aimed at investigating the effect of phospholipids on the CLA of PDC-109. Vesicles were prepared by taking a small amount (∼0.5 mg) of the appropriate phospholipid in a glass test tube, dissolving it in ca. 100–200 µL of dichloromethane or dichloromethane/methanol mixture, followed by evaporation of the solvent under a continuous stream of nitrogen gas and vacuum desiccation. The lipid film thus obtained was hydrated with an appropriate volume of TBS-I to give the desired concentration of phospholipid (∼1 mM) and then subjected to bath sonication (30–60 minutes), which yielded a transparent solution, indicating the formation of unilamellar vesicles. To investigate the effect of phospholipid binding on the protein conformation, PDC-109 (1.0 mg/mL) was incubated with unilamellar vesicles of DMPC or DMPG (final concentration: 0.1 mM) for 5 minutes and then CD spectra were recorded at room temperature on a Jasco J-810 spectropolarimeter, essentially as described above.

Chaperone activity was assessed by standard aggregation assays, where heat induced aggregation of target proteins [LDH, ADH and carbonic anhydrase (CA)] was monitored as light scattering, as a function of time. To study the effect of phospholipid binding on the CLA of PDC-109, individual phospholipids (0–2 µM) were mixed with a fixed concentration of PDC-109 and allowed to bind for 5 minutes. LDH was pre-incubated with the appropriate PDC-109/phospholipid mixture for 5 minutes and then placed in a spectrophotometer cuvette to record the light scattering of the solutions. Aggregation of native LDH was taken as 100% and the aggregation of other samples was normalized with respect to that of the native enzyme. Concentrations of PDC-109, target proteins and the lipids as well as the temperature used in different assays are given in the captions to the figures presenting the results of the experiments.

### BisANS binding to PDC-109, phospholipid vesicles and PDC-109/phospholipid mixtures

Interaction of bisANS with native PDC-109, phospholipid vesicles (DMPC or DMPG) and PDC-109/phospholipid mixtures was investigated at 25°C by steady-state fluorescence spectroscopy. Unilamellar vesicles of DMPC and DMPG were prepared by bath sonication as described above. The following samples were prepared in TBS-I: bisANS; bisANS + PDC-109; bisANS + DMPC; bisANS + DMPG; bisANS + DMPC + PDC-109; bisANS + DMPG + PDC-109. The concentrations of PDC-109, phospholipid and bisANS in individual samples were fixed at 0.05 mg/mL, 5 µM and ∼12 µM, respectively. For the last two samples, PDC-109 and phospholipid vesicles were mixed and allowed to stand at room temperature for 10 minutes before the addition of bisANS. Fluorescence measurements were performed on a Spex-Fluoromax-4 fluorescence spectrometer. Samples were excited at 385 nm and emission spectra were recorded between 395 and 650 nm. Slit widths of 3 and 5 nm were used on the excitation and emission monochromators, respectively.

### Effect of cholesterol incorporation into the phospholipid membrane, on the CLA

Unilamellar vesicles of DMPC and DMPC/cholesterol (4∶1, mol/mol) were prepared by bath sonication as described above. CA was used as the target protein and the CLA was monitored by using standard aggregation assays, where heat induced aggregation of the target protein was recorded as light scattering at 360 nm. In a typical experiment, 0.2 mg/mL of CA was incubated with a fixed concentration of PDC-109 (0.2 mg/mL) for 5 minutes at room temperature and the mixture was then subjected to heat stress by incubating for one hour at 52°C. To investigate the effect of cholesterol incorporation into DMPC vesicles on the CLA of PDC-109, mixtures of DMPC and PDC-109, or DMPC, cholesterol and PDC-109 (containing 5 µM of the respective phospholipid and 0.2 mg/mL of PDC-109) were pre-incubated with CA for 5 minutes and then the assay was performed in a similar way as described above. Aggregation profiles of the two mixtures were compared in order to assess the effect of the cholesterol incorporation on the CLA of PDC-109.

### Effect of DMPC and DMPC/cholesterol mixture on the CLA of PDC-109: AFM studies

Carbonic anhydrase was used as a target protein in the AFM studies aimed at investigating the effect of DMPC or DMPC/cholesterol on the CLA of PDC-109. Samples of native CA (0.2 mg/ml) and CA-PDC-109 mixture (1∶1 ratio, w/w) were prepared by incubating them at 52°C in a dry bath for 1 hour. To probe the effect of DMPC or DMPC/cholesterol binding on the CLA of PDC-109, a mixture containing CA, PDC-109 and DMPC (or DMPC/cholesterol; 4∶1, mol/mol) was prepared and incubated at 52°C for 1 hour. Here, PDC-109 was first incubated with DMPC or DMPC/cholesterol mixture for 5 minutes and then CA was added to obtain the ternary mixture. The final concentrations of CA, PDC-109 and DMPC in the above mixture were 0.2 mg/mL, 0.2 mg/mL and 5 µM, respectively. A 20–30 µL aliquot of the respective sample was carefully deposited on a freshly cleaved mica sheet (1 cm×1 cm) and allowed to dry for 20–30 minutes, rinsed with HPLC grade water, dried and transferred to the AFM stage. Imaging was performed in semi-contact mode using a SOLVER PRO-M atomic force microscope (NT-MDT, Moscow, Russia), equipped with a 10.0 µm bottom scanner. NSG10 cantilevers with Au reflective coating and a nominal spring constant of 11.8 N/m were used for the scanning. Force was kept at the lowest possible value by continuously adjusting the set-point and feed-back gain during imaging.

## Results and Discussion

In an earlier study we have demonstrated that PDC-109 exhibits chaperone-like activity and that the polydisperse nature of PDC-109 is critical for its CLA [23]. However, a knowledge of other factors that are important for such an activity, or modulate the CLA, is still lacking. In the present study we have carried out additional experiments to characterize the CLA of PDC-109 using two more target proteins, namely aldolase and carbonic anhydrase. In addition, the effect of phospholipid binding on the CLA of PDC-109 and the role of hydrophobicity in it were also investigated.

### Chaperone-like activity of PDC-109

**Figure 1:**
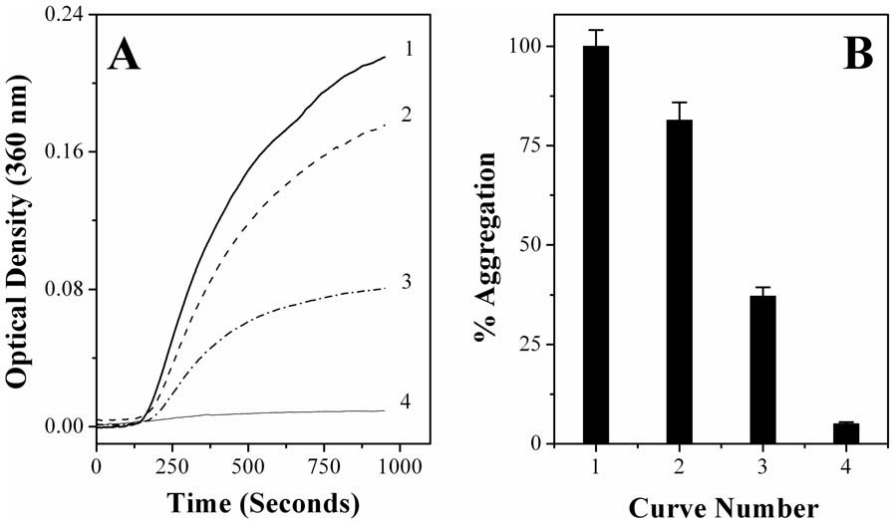
Prevention of thermal aggregation of aldolase by PDC-109. Concentration of aldolase was 0.93 µM in all samples. **A**) Aggregation profiles recorded at 48°C are shown for aldolase alone (curve 1), aldolase + 0.39 µM PDC-109 (curve 2), aldolase + 0.78 µM PDC-109 (curve 3) and aldolase + 1.44 µM PDC-109 (curve 4). **B**) Bar diagram representing percent aggregation of aldolase (at 900 seconds) under different conditions.

Results of turbidimetric studies aimed at investigating the effect of PDC-109 on the thermal aggregation of aldolase are shown in Fig. 1A. The results indicate that incubation of aldolase at 48°C leads to a rapid increase in the sample turbidity initially, which slows down later on, displaying saturation behavior (curve 1). Presence of PDC-109 reduced the rate of this aggregation significantly in a concentration-dependent manner. At a PDC-109 to aldolase ratio of 1∶2.4 the extent of aggregation decreased to 81.4% as compared to that of the native enzyme at the end point of the assay (curve 2). The aggregation decreased to 37.2% when the PDC-109 to aldolase ratio was increased to 1∶1.2 (curve 3), whereas just 5% aggregation was observed at a ratio of 1∶0.6 (curve 4). A bar diagram showing percent aggregation versus concentration of PDC-109 is given in Fig. 1B. Qualitatively very similar results were obtained when carbonic anhydrase was used as the target protein (Fig. S1). Prevention of heat induced aggregation of aldolase and CA by PDC-109 lends further support to our previous report that PDC-109 exhibits chaperone-like activity against a variety of target proteins [23].

### Isothermal titration calorimetric studies on bisANS binding to PDC-109

**Figure 2:**
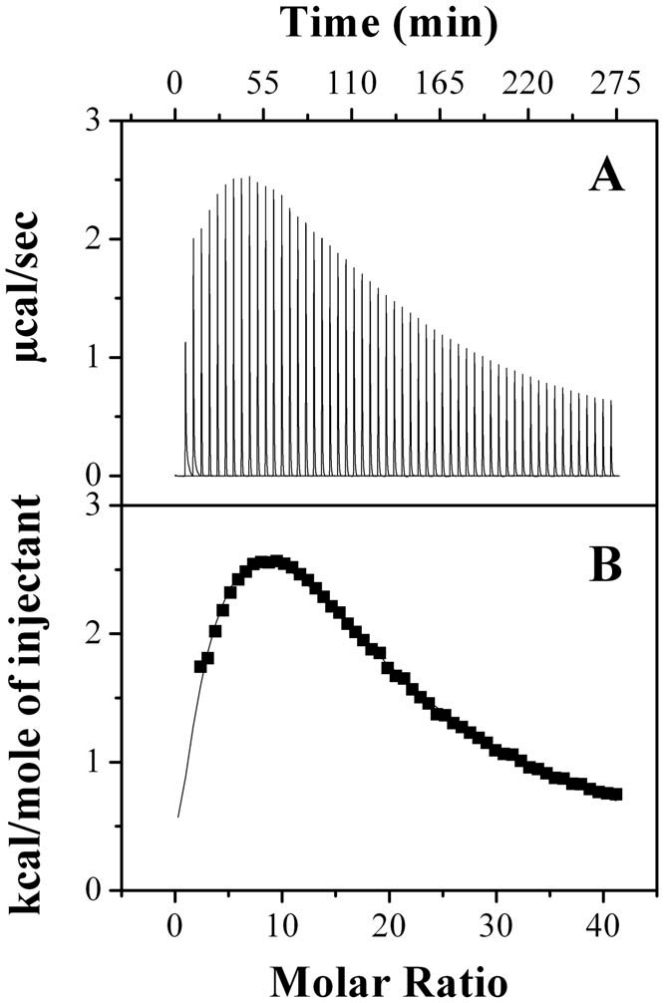
Calorimetric titration of bisANS binding to PDC-109. Raw data for the titration of bisANS with PDC-109 at 30°C is shown in the upper panel (**A**) and the integrated heats of binding obtained from the raw data are shown in the lower panel (**B**), after subtracting the heat of dilution. The solid line in the lower panel represents the best fit of the experimental data to the *sequential binding model* in the MicroCal Origin program. See text for further details.

ITC measurements to investigate the binding of bisANS to PDC-109 were carried out at 30°C and a typical ITC profile is shown in Fig. 2. From this figure it can be seen that the binding process is endothermic in nature. The titration data could be best fitted using a *sequential binding model*, which suggests the presence of two types of binding interactions between bisANS and PDC-109 (Fig. 2). Thermodynamic parameters obtained from this analysis, listed in Table 1, indicate that both the interactions are endothermic in nature with positive changes in enthalpy and large positive entropic contributions, which compensate for the unfavorable enthalpy term and yield a net negative value of free energy (Δ*G*). We found that the final values of enthalpy and entropy associated with each binding site varied somewhat depending on the input values, although overall both enthalpy and entropy remained positive irrespective of the input data. Also, the binding constants did not exhibit much variance. The results obtained with one of the good fits are summarized in Table 1, which show that both the binding sites have similar affinity for bisANS. Variation in the binding parameters depending on the method used for bisANS interaction with other proteins has also been reported [44]. The entropy-favored binding of bisANS to PDC-109 suggests the presence of hydrophobic patches on the protein surface, which in turn could be useful in its interaction with the non-native structures of different target proteins, which prevents their aggregation under stress conditions.

**Table 1:** Thermodynamic parameters for the interaction of bisANS with PDC-109.

Binding Site	*K_a_* × 10^3^ (M^−1^)	−Δ*G* kcal.mol^−1^	Δ*H* kcal.mol^−1^	Δ*S* cal.mol^−1^.K^−1^
N1	2.57±0.1	4.71±0.6	7.8±1.02	41.3
N2	2.53±0.07	4.65±0.07	101.4±1.5	350

The values of the parameters were obtained by analyzing the data obtained from isothermal titration calorimetry to a sequential binding model. Values shown correspond to the averages obtained from two independent titrations and standard deviations are indicated.

### ANS binding to PDC-109 and hydropathy indexing

**Figure 3:**
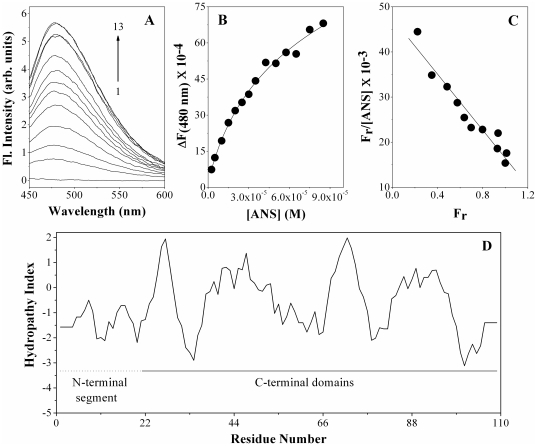
Fluorescence titration for ANS binding to PDC-109. **A**) Fluorescence spectra of ANS binding to PDC-109. Spectrum 1 corresponds to PDC-109 alone and spectra 2–13 were recorded in the presence of increasing concentrations of ANS. **B**) Binding curve obtained by plotting change in fluorescence intensity (ΔF) as function of ANS concentration. **C**) Linear Scatchard plot for the binding data. Slope of this plot gives association constant for the interaction and the X-intercept yields the stoichiometry of binding. **D**) Hydropathy plot of PDC-109 according to Kyte & Doolittle (1982) using using a 7-residue window. See text for details.

Fluorescence spectra and binding curve corresponding to the titration of PDC-109 with ANS are given in Figs. 3A & 3B, respectively. It is seen from these two figures that addition of ANS to PDC-109 leads to an increase in the fluorescence intensity of the probe and the magnitude of the change decreases with increasing ANS concentration, depicting saturation behavior. In these experiments, PDC-109 concentration was kept very low in order to maintain [ANS]_bound_ << [ANS]_total_, such that for further analysis of the titration data, [ANS]_total_ could be used as a good approximation of free ligand concentration [45], [46]. From the titration data the association constant for the binding of ANS to PDC-109 was estimated by Scatchard analysis [45], [46]. From the slope of the Scatchard plot, given in Fig. 3C, the association constant, *K*_a_ was estimated as 3.09×10^4^ M^−1^, whereas the X-intercept of the plot was obtained as 1.2, indicating that each molecule of PDC-109 has one binding site for ANS, which is comparable with ANS binding to α-crystallin [47], [48].

Hydropathy analysis performed according to Kyte and Doolittle [49] with a sliding window of 7 residues suggests that the sequence corresponding to the two FnII domains of PDC-109 is more hydrophobic than the preceding N-terminal segment (Fig. 3D). Although this does not necessarily imply that the FnII domains are directly responsible for the CLA, it is possible that interaction of apolar residues of these domains with target proteins may be relevant to the CLA of PDC-109.

### Effect of ANS binding on the CLA and secondary structure of PDC-109

**Figure 4:**
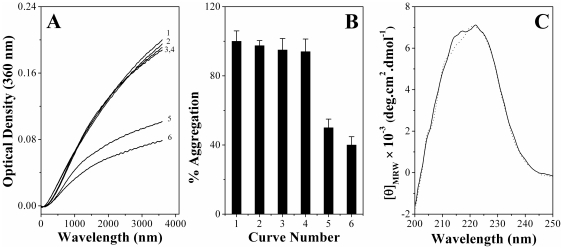
Effect of ANS binding on the CLA and secondary structure of PDC-109. **A**) Prevention of aggregation of LDH (0.15 mg/ml) by PDC-109. Aggregation profiles shown correspond to the following samples incubated at 48°C: (1) LDH, (2) LDH + 6 µM ANS, (3) LDH + 0.05 mg/ml PDC-109, (4) LDH + 0.05 mg/ml (ANS-PDC-109), (5) LDH + 0.075 mg/ml of PDC-109 and (6) LDH + 0.075 mg/ml of (ANS-PDC-109). **B**) Bar diagram representing percent aggregation of LDH under different conditions (at 3600 seconds) as shown in (**A**). **C**) Far UV CD spectra of PDC-109 in the absence (solid line) and presence (dotted line) of ANS at room temperature.

Aggregation assays aimed at investigating the effect of ANS binding on the CLA of PDC-109 are shown in Fig. 4A. Incubation of LDH at 48°C results in a rapid increase in the turbidity of the sample with time in the initial stages, which slows down afterwards (curve 1). A PDC-109 to LDH ratio (w/w) of 1∶3 led to a 5% reduction in the extent of aggregation at 60 minutes (curve 3), whereas aggregation decreased further to 50% when the PDC-109/LDH ratio (w/w) was increased to 1∶2 (curve 5). ANS binding did not lead to any significant change in the CLA at a PDC-109-ANS:LDH ratio of 1∶3 (curve 4), although a slight increase in the CLA (about 10%) was observed, as compared to native PDC-109, when the ratio was increased to 1∶2 (curve 6). The protein fractions eluted from the gel filtration column showed a molar ratio of 1∶1 for the PDC-109 and bound ANS. ANS alone had a negligible effect on the aggregation of target protein at the concentrations mentioned above (curve 2). A bar diagram, indicating the percent aggregation of LDH under different conditions is shown in Fig. 4B.

Far UV CD spectra, shown in Fig. 4C, indicate that the secondary structure of PDC-109 in the presence of ANS (dotted line) is nearly identical to that of the native protein (solid line), suggesting that ANS binding does not result in any significant changes in the conformation of the protein. This could be one reason why ANS binding does not have any noticeable effect on the CLA of PDC-109. Similar results were observed using ADH as the target protein and the results obtained are shown in Fig. S2.

### Phospholipid induced modulation of the CLA of PDC-109 and alterations in its tertiary structure

**Figure 5:**
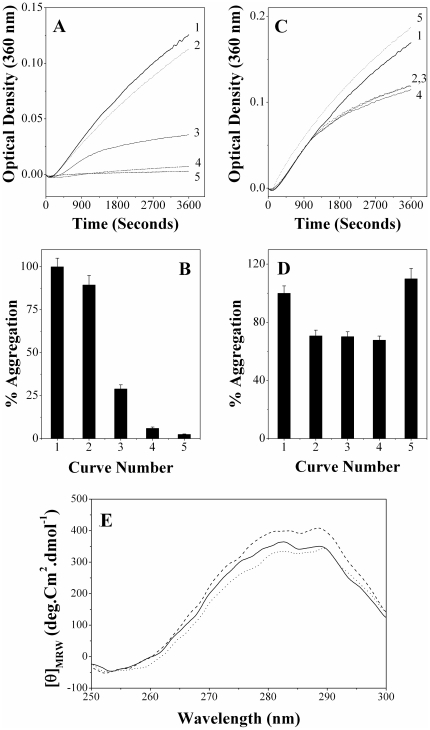
Effect of phospholipid binding on the CLA and tertiary structure of PDC-109. **A**) Effect of DMPC on the prevention of aggregation of LDH by PDC-109. Aggregation profiles shown correspond to the following samples incubated at 48°C: (1) LDH alone, (2) LDH +2 µM of DMPC, (3) LDH + PDC-109, (4) LDH + PDC-109 + DMPC (0.5 µM) and (5) LDH + PDC-109 + DMPC (2 µM). In these experiments LDH and PDC-109 were used at a concentration of 0.1 and 0.075 mg/ml, respectively. **B**) Bar diagram representing percent aggregation of LDH for different samples shown in panel (**A**) at 3600 seconds. **C**) Effect of DMPG on the prevention of aggregation of LDH by PDC-109. Aggregation profiles shown correspond to the following samples incubated at 48°C: (1) LDH alone, (2) LDH + PDC-109 + DMPG (0.1 µM), (3) LDH + PDC-109 + DMPG (0.5 µM), (4) LDH + PDC-109 and (5) LDH + DMPG (0.5 µM). In these experiment LDH and PDC-109 were used at a concentration of 0.12 and 0.05 mg/ml, respectively. **D**) Bar diagram for the data shown in (**C**) at 3600 seconds. **E**) Near UV CD spectra of PDC-109 under native conditions (solid line), in the presence of DMPC (dashed line) and DMPG (dotted line).

Results of aggregation assays to investigate the effect of phospholipid binding on the CLA of PDC-109 are shown in Fig. 5. LDH, which is an aggregation-prone enzyme, was used as the target protein. Upon incubation at 48°C, turbidity of the sample containing LDH increases rapidly with time initially, but slows down with further incubation (curve 1, Fig. 5A). In the presence of PDC-109 at a PDC-109:LDH ratio of 3∶4, the extent of aggregation of LDH decreased to ∼29% with respect to that of the native enzyme (curve 3, Fig. 5A). Pre-incubation of PDC-109 with 0.5 µM DMPC (unilamellar vesicles) resulted in a significant decrease in the aggregation and at the same PDC-109:LDH ratio, only 6% aggregation was observed as compared to that of the native enzyme, whereas no aggregation was seen when PDC-109 was pre-incubated with 2 µM DMPC (curves 4 & 5, Fig. ;5A).

On the other hand, inclusion of DMPG vesicles (0.1 and 0.5 µM) in the solution did not yield any alteration in the CLA (curves 2 & 3, Fig. 5C), which was comparable to the CLA of PDC-109 alone (curve 4). The concentrations of LDH and PDC-109 used in this experiment are mentioned in the figure caption (Fig. 5C). Neither DMPC nor DMPG significantly affected the aggregation profiles of target protein at the above concentrations in the absence of PDC-109 (curve 2, Fig. 5A & curve 5, Fig. 5C). Similar results were obtained in the experiments performed using CA and ADH as target proteins (see Figs. S1 and S3). Near-UV CD spectra of PDC-109 obtained in the presence of these two phospholipids show considerable differences, when compared with the spectrum of native PDC-109 (Fig. 5E), indicating that the protein tertiary structure is altered upon phospholipid binding, which is consistent with earlier reports [15], [39].

The dramatic change in the CLA of PDC-109, observed in the presence of DMPC can be explained in two different ways. One explanation is that the structural changes in PDC-109, induced by its interaction with DMPC, can modulate the CLA. Results of our CD studies are in agreement with this (Fig. 5E). The other explanation is based on PDC-109 induced partial solubilization of the vesicles and formation of lipid/protein particles of various sizes, which could modulate the overall hydrophobicity of the environment, which may alter the CLA. Our parallel ITC studies on the binding of PDC-109 to DMPC model membranes showed that the interaction is characterized by a large positive heat capacity change (Δ*C*_p_), which suggested that large hydrophobic surfaces are exposed to water upon binding of PDC-109 to DMPC membranes [50]. Increase in the hydrophobicity upon interaction of PDC-109 with DMPC vesicles, was also probed by studying ANS and bisANS binding as discussed below. As shown above, another phospholipid, i.e., phosphatidylglycerol, had only a marginal influence on the CLA of PDC-109 (Fig. 5C), which could be attributed to the low affinity of its binding to PDC-109 as compared to phosphatidylcholine.

PDC-109 has been reported to play an important role in priming spermatozoa for fertilization by a membrane remodeling process [9] and in a previous report we have demonstrated that PDC-109 can also function as a molecular chaperone, which could be of physiological significance during fertilization [23]. The present results establish a correlation between the membrane binding and chaperone-like activity of PDC-109, and therefore are of considerable relevance for the better understanding of the role of PDC-109 in fertilization.

### Enhanced surface hydrophobicity upon interaction of PDC-109 with phosphatidylcholine membrane

**Figure 6:**
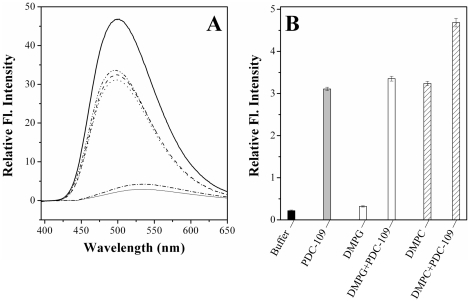
Binding of bisANS to phospholipids, PDC-109 and phospholipid-PDC-109 mixtures. (**A**) Fluorescence spectra shown correspond to: bisANS in buffer (solid thin line), DMPG (5 µM, dash-dot line), PDC-109 (0.05 mg/ml, dotted line), DMPC (5 µM, dashed line), DMPG-PDC-109 mixture (dash-dot-dot line) and DMPC-PDC-109 mixture (solid thick line). The final concentration of bisANS in each of the sample is 12.2 µM. Relative fluorescence intensity of different samples is shown in the form of a bar diagram in panel (**B**).

BisANS is a fluorescent probe that is being used extensively for identifying and characterizing hydrophobic regions of proteins and other biomolecules. Upon binding to such sites it displays enhanced fluorescence intensity [51]. Therefore, the extent of enhancement in its fluorescence intensity could be correlated to increased exposure of hydrophobic residues or surfaces. Fluorescence spectra corresponding to bisANS alone and in the presence of PDC-109, DMPC, DMPG, and mixtures of PDC-109 with DMPC and DMPG are shown in Fig. 6A. Fluorescence enhancement upon bisANS binding to DMPG vesicles (dash-dot line), was comparable to that of the control sample (buffer + bisANS, solid thin line), whereas a 11.3-fold enhancement was observed upon binding to DMPC (dash line), which is comparable to the fluorescence enhancement observed upon binding to native PDC-109 (dot line). Fluorescence enhancement upon bisANS binding to DMPG-PDC-109 mixture was slightly higher than that observed for binding to native PDC-109 (dash dot dot line), whereas it was nearly 1.5-fold higher as compared to the enhancement observed for binding to native PDC-109 and 16.4-fold higher than control (buffer + bisANS), in the case of DMPC-PDC-109 complex (solid thick line). A bar diagram indicating the relative fluorescence intensity of bisANS in various samples is shown in Fig. 6B.

Dramatic increase in the fluorescence intensity of bisANS, upon binding to DMPC-PDC-109 mixture indicates increased exposure of hydrophobic surfaces. Since PDC-109 binds to choline phospholipids with high affinity, which leads to membrane disruption and formation of lipid/protein complexes, the above results indicate that these lipid/protein recombinants have a higher degree of surface hydrophobicity. The increased hydrophobicity of these recombinants may be important for their better interaction with partially unfolded target proteins as compared to PDC-109 alone and prevention of their aggregation. This interpretation is consistent with the enhanced chaperone-like activity of DMPC-PDC-109 mixtures as compared to PDC-109 alone (Figs. 5A, S1A and S3A). PDC-109 has a significantly lower affinity for phosphatidylglycerol as compared to phosphatidylcholine [18], [21] and hence its binding to DMPG membranes may not expose the interior hydrophobic regions of this lipid to the same extent as is observed upon its binding to DMPC membranes. The lack of significant change in the fluorescence intensity of bisANS (dash dot dot line, Figs. 6A) as well as in the CLA of the protein (Figs. 5C, S1C and S3C) in the presence of DMPG-PDC-109 mixture, are consistent with the above interpretation. Similar results were obtained for ANS binding to the samples described above and the results obtained are shown in Fig. S4.

Alteration in the hydrophobicity of PDC-109 upon binding of PrC was also investigated by ANS binding (Text S1, Fig. S5). These experiments indicated that PrC binding leads to a significant decrease in the surface hydrophobicity of the protein (Text S1). In our earlier study the dramatic decrease in the CLA of PDC-109 upon PrC binding was attributed to the dissociation of the polydisperse aggregates of the protein [23]. The present results indicated that in addition to polydispersity, surface hydrophobicity is also important for the CLA of PDC-109.

### Cholesterol incorporation into phospholipid vesicles modulates the CLA of PDC-109

**Figure 7:**
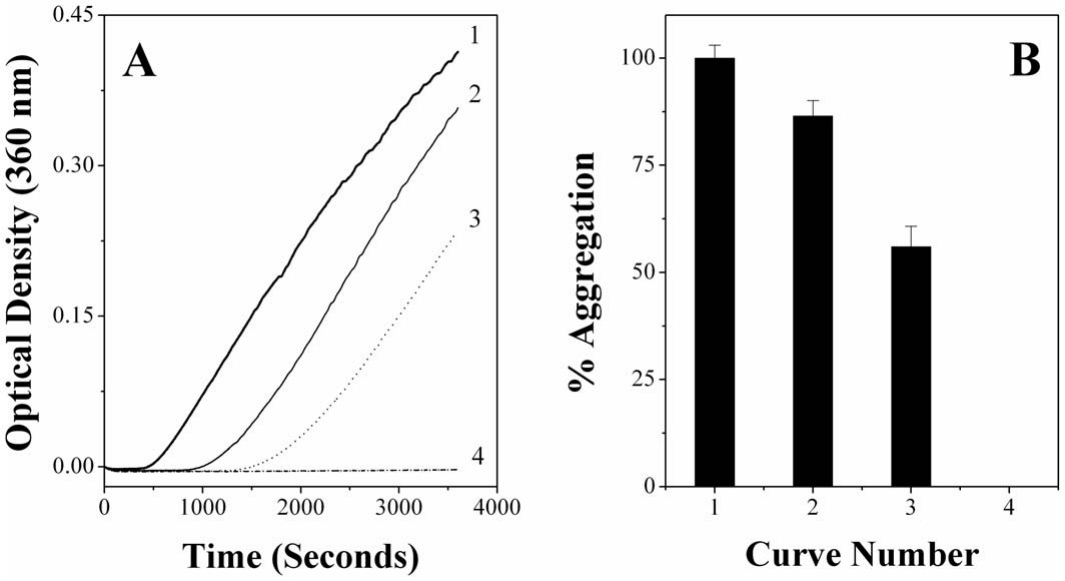
Effect of cholesterol incorporation into the phospholipid membrane on the CLA of PDC-109. (**A**) Prevention of aggregation of CA. Aggregation profiles shown correspond to the following samples incubated at 52°C: (1) CA alone, (2) CA + PDC-109, (3) CA + PDC-109 + DMPC/cholesterol and (4) CA + PDC-109 + DMPC. Concentrations of both CA and PDC-109 were 0.2 mg/ml in all the samples. **B**) Bar diagram representing percent aggregation of CA under different conditions as shown in panel (**A**) at 3600 seconds.

As discussed above, incubation of PDC-109 with DMPC unilamellar vesicles leads to dramatic alteration in the aggregation behavior of various target proteins (Fig. 5A, S1A and S3A). In order to investigate the effect of cholesterol, similar experiments were carried out with samples containing 25 mol% cholesterol in DMPC unilamellar vesicles and the results obtained are shown in Fig. 7. Incubation of CA at 52°C results in a rapid increase in the turbidity which reaches a maximum and slows down with time (curve 1, Fig. 7A). The extent of aggregation of CA at the end point of assay (60 minutes) was considered as 100% and the aggregation of other samples was normalized with respect to this. Prior incubation of CA with PDC-109 led to about 15% reduction in the aggregation (curve 2), whereas pre-incubation of CA with DMPC-PDC-109 mixture resulted in a complete inhibition of aggregation (curve 4). However, pre-incubation with DMPC/cholesterol-PDC-109 mixture resulted in only 45% inhibition of CA aggregation (curve 3), indicating that cholesterol incorporation into DMPC vesicles has a negative impact on the CLA of PDC-109/DMPC recombinants (Figs. 5A, S1A and S3A). However, it must be noted that DMPC/cholesterol-PDC-109 recombinants exhibited higher CLA than PDC-109 alone. Similar results were obtained when LDH was used as the target protein (see Fig. S6).

Upon binding to phospholipid membranes, PDC-109 removes lipid molecules from the outer leaflet [10], [11], [52], leading to membrane disruption. Our fluorescence studies of bisANS and ANS binding to DMPC-PDC-109 mixtures suggest that surface hydrophobicity of the PDC-109/DMPC recombinants is significantly higher as compared to that of PDC-109 alone (Figs. 6 and S4). It is known that the interaction of PDC-109 with membranes containing phosphatidylcholine membranes results in the formation of lipid/protein particles with high curvature [16]. In such highly curved aggregates of lipids, the hydrophobic regions of the lipids will be more exposed than in normal lipid bilayers. Also, conformational changes in the protein resulting from lipid binding [15] may also lead to increased exposure of hydrophobic regions of the protein. Most probably these additional hydrophobic surfaces interact with the non-native structures of aggregation prone proteins, resulting in their protection under stress conditions. On the other hand, interaction of PDC-109 with DMPC vesicles containing 25 mol% cholesterol could not extend similar protection to the target proteins (Figs. 7A and S6). Since cholesterol is known to provide rigidity to membranes, PDC-109 binding cannot induce the complete disruption of such vesicles [15], [18], [21]. This interaction therefore cannot lead to the exposure of the hydrophobic surfaces to the same extent as is achieved by the interaction of PDC-109 with membranes containing only phosphatidylcholine. The CLA of PDC-109 in the presence of DMPC/cholesterol mixtures is therefore relatively less as compared to that of PDC-109/DMPC recombinants. It is also possible that direct interaction of cholesterol with the CRAC domain of PDC-109 may alter the CLA of the protein [53].

### Modulation of the CLA of PDC-109 by DMPC or DMPC/cholesterol mixture: AFM studies

**Figure 8:**
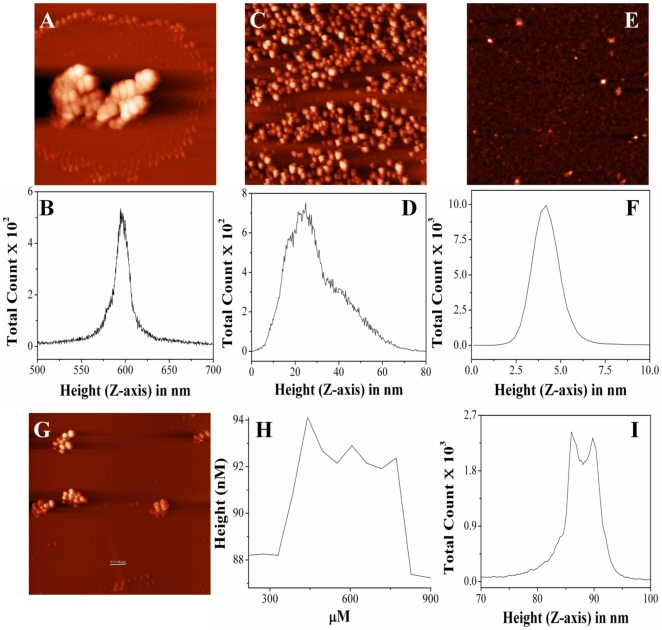
AFM studies on the modulation of CLA of PDC-109 by cholesterol incorporation into DMPC membranes. The ability of cholesterol to modulate the chaperone-like activity of PDC-109/DMPC recombinants was investigated by AFM using carbonic anhydrase as the target protein. (**A**) AFM image of CA upon incubation at 52°C (14×14 µm). (**B**) Distribution density histogram for the image shown in (**A**). (**C**) AFM image of CA upon incubation at 52°C in the presence of PDC-109 (5×5 µm). (**D**) Distribution density histogram for the image shown in (**C**). (**E**) AFM image of CA upon incubation at 52°C in the presence of DMPC-PDC-109 (6×6 µm). (**F**) Distribution density histogram for the image shown in (**E**). (**G**) AFM image of CA upon incubation at 52°C in the presence of DMPC/cholesterol-PDC-109 (14×14 µm). (**H**) Height profile corresponding to the bar shown in image (**G**). (**I**) Distribution density histogram for the image shown in (**G**).

**Figure 9:**
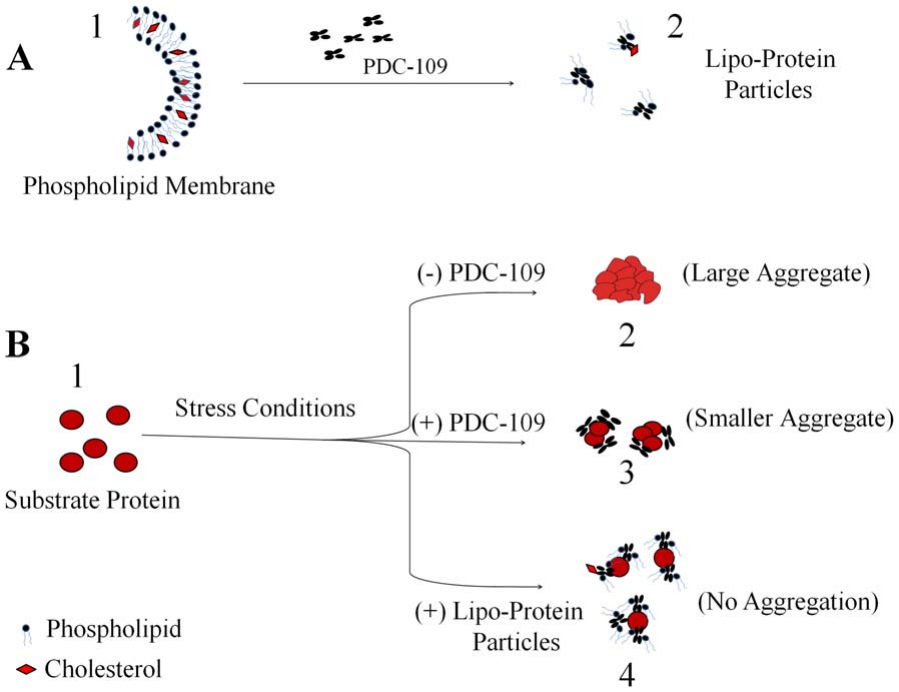
A schematic model for the functional role of PDC-109 in sperm capacitation. **A**) PDC-109 binding induces cholesterol and choline phospholipids efflux, which is a necessary step for sperm capacitation. Phospholipid membrane (1) and efflux particles made up of PDC-109, cholesterol and choline phospholipids (2), are shown. **B**) PDC-109 functions as a molecular chaperone and stabilizes substrate proteins. Substrate proteins (1), are aggregated under stress conditions (2), which in the presence of PDC-109 (3) or lipid/protein recombinants (4) are stabilized efficiently.

The effect of DMPC or DMPC/cholesterol binding on the CLA of PDC-109 was also studied using AFM, essentially as described earlier [23]. AFM images of CA, obtained after incubation at 52°C indicate that this enzyme forms very large aggregates of several microns in size upon such treatment (Fig. 8A). However, when CA was incubated at the same temperature in the presence of PDC-109, considerably smaller aggregates were formed under similar conditions (Fig. 8C). Distribution density histogram shows a size distribution in the range of 550–650 nm for CA alone (at 52°C), which shifted to 10–60 nm in the presence of PDC-109 (Figs. 8B & D). This shows that although PDC-109 prevents the aggregation of CA but is not as efficient as it is against LDH and ADH [23]. Interestingly, PDC-109 pre-incubated with DMPC prevented the aggregation of CA significantly and the size distribution was observed to be <10 nm (Figs. 8E & F). This is consistent with our turbidimetric studies where inclusion of DMPC with PDC-109 resulted in multifold enhancement of its CLA (Figs. 5A, S1A and S3A). On the other hand, pre-incubation of PDC-109 with DMPC vesicles containing 25 mol% cholesterol did not prevent the aggregation of CA as efficiently as DMPC alone (Fig. 8G). As discussed above, the enhanced CLA upon interaction of PDC-109 with DMPC vesicles appears to be due to the increased exposure of hydrophobic surfaces upon such interaction. The presence of a small membrane segment in Fig. 8G is consistent with the ability of cholesterol to stabilize choline phospholipid membranes against PDC-109-induced disruption, due to which the exposure of hydrophobic surfaces is decreased. This, in turn, would result in a decrease in the CLA of PDC-109/DMPC recombinants in the presence of cholesterol. These observations demonstrate that the lipid/protein recombinants, formed by the interaction of PDC-109 with phospholipid membranes, exhibit higher CLA and prevent the aggregation of substrate proteins more efficiently than PDC-109 alone. This is schematically illustrated in Fig. 9.

### Conclusions

In summary, our *in vitro* studies demonstrate that the lipid/protein complexes formed by the high-affinity binding of PDC-109 to membranes containing choline phospholipids, exhibit a higher chaperone-like activity than PDC-109 alone. The lipid/protein recombinants possess a higher surface hydrophobicity as compared to native PDC-109, which could be responsible for their better interaction with partially unfolded proteins and prevention of their aggregation under stress conditions. Cholesterol incorporation resulted in a decrease in the CLA of the PDC-109/DMPC recombinants, which could be attributed to membrane rigidification, or to direct interaction of cholesterol with the CRAC domain of PDC-109. However, the CLA of lipid/protein complexes was always higher than that of PDC-109 alone. ANS binding to PDC-109 in the presence of PrC suggests that in addition to polydispersity, hydrophobicity is also an important factor for the CLA of PDC-109. The observation of CLA by PDC-109 in the presence of phospholipids also mimics the *in vivo* environment where PDC-109 could bind to the outer leaflet of the sperm plasma membrane, which makes the experiments of this study physiologically relevant. The present study could also be important to understand the functional significance of membrane/protein interaction for the other chaperonins such as spectrin and Hsp12 [54], [55]. Overall our present results shed further light on the CLA of PDC-109 and demonstrate that hydrophobicity plays an important role in this activity.

## Supporting Information

Figure S1 **Effect of phospholipid binding on the CLA of PDC-109.** **A**) Prevention of aggregation of CA (0.2 mg/ml) by PDC-109. Aggregation profiles of (1) Native CA at 52°C, (2) CA + 0.2 mg/ml PDC-109, (3) CA +5 µM of DMPC, (4) CA + PDC-109 (0.2 mg/ml) + DMPC (2 µM) and (5) CA + PDC-109 (0.2 mg/ml) + DMPC (5 µM) are shown. **B**) Bar diagram representing percent aggregation of CA under different conditions as shown in (**A**) at 3600 seconds. **C**) Aggregation profiles of (1) Native CA at 52°C, (2) CA + 0.2 mg/ml PDC-109, (3) CA + DMPG (2 µM) and (4) CA + PDC-109 (0.2 mg/ml) + DMPG (2 µM) are shown. **D**) Bar diagram for the data shown in (**C**) at 3600 seconds.
(TIF)

Figure S2 **Effect of ANS binding on the CLA of PDC-109.** Modulation of CLA of PDC-109 by ANS binding was investigated by aggregation assay with ADH (0.1 mg/mL) as the target protein. **A**) Aggregation profiles of (1) ADH at 48°C, (2) ADH + 0.025 mg/ml PDC-109, (3) ADH + 0.025 mg/ml ANS-PDC-109, (4) ADH + 0.05 mg/ml of PDC-109 and (5) ADH + 0.05 mg/ml of ANS-PDC-109 are shown. **B**) Bar diagram representing percent aggregation of LDH under different conditions as shown in (**A**) at 960 seconds.
(TIF)

Figure S3 **Effect of phospholipid binding on the CLA of PDC-109.** Prevention of aggregation of ADH (0.05 mg/ml) by PDC-109. **A**) Aggregation profiles of (1) Native ADH at 48°C, (2) ADH + 2 µM of DMPC, (3) ADH + PDC-109 (0.025 mg/ml) and (4) ADH + PDC-109 (0.025 mg/ml) + DMPC (2 µM) are shown. **B**) Bar diagram representing percent aggregation of ADH under different conditions as shown in panel (**A**) at 960 seconds. **C**) Aggregation profiles of (1) Native ADH at 48°C, (2) ADH + DMPG (0.1 µM), (3) ADH + PDC-109 (0.03 mg/ml), (4) ADH + PDC-109 (0.03 mg/ml) + DMPG (0.05 µM) and (5) ADH + PDC-109 (0.03 mg/ml) + DMPG (0.1 µM) are shown. **D**) Bar diagram for the data shown in (**C**) at 900 seconds.
(TIF)

Figure S4 **ANS Binding to phospholipids, PDC-109 and phospholipid-PDC-109 mixtures.** **A**) Fluorescence spectra for the interaction of ANS with buffer (solid thin line), DMPG (5 µM, dash dot line), PDC-109 (0.05 mg/ml, dot line), DMPC (5 µM, dash line), DMPG-PDC-109 mixture (dash dot dot line) and DMPC-PDC-109 mixture (solid thick line) are shown. Final concentration of ANS in each sample was 50 µM. **B**) Relative fluorescence intensity of different samples at the emission maximum.
(TIF)

Figure S5 **ANS Binding to PrC and PrC-PDC-109 mixtures.** **A**) Fluorescence spectra of ANS in TBS-1 under different conditions. 1) with PrC; 2) with PDC-109 + PrC; 3) with PDC-109. Concentrations of different components used were: ANS, 50 µM; PDC-109, 0.05 mg/mL; PrC, 10 mM. **B**) Relative fluorescence intensity of different samples at the emission maximum.
(TIF)

Figure S6 **The Effect of cholesterol incorporation into phospholipid membrane, on the CLA of PDC-109.** **A**) Prevention of aggregation of LDH (0.15 mg/ml). Aggregation profiles of (1) Native LDH at 50°C, (2) LDH + 0.075 mg/ml PDC-109, (3) LDH + PDC-109 (0.075 mg/ml) + DMPC/cholesterol (2 µM) and (4) LDH + PDC-109 (0.075 mg/ml) + DMPC (2 µM) are shown. **B**) Bar diagram representing percent aggregation of LDH under different conditions as shown in (**A**) at 3600 seconds.
(TIF)

Text S1 **Methods and Results.** Experimental details employed for studying the binding of ANS to PDC-109 and PDC-109/PrC complex and the results obtained from these experiments are provided.
(DOC)

## References

[1] Shivaji S, Scheit KH, Bhargava PM (1990). Proteins of seminal plasma..

[2] Yanagimachi R, Knobil E, Neill JD (1994). Mammalian fertilization..

[3] Chang MC (1951). Fertilizing capacity of spermatozoa deposited into the fallopian tubes.. Nature.

[4] Manjunath P, Sairam MR, Uma J (1987). Purification of four gelatin-binding proteins from bovine seminal plasma by affinity chromatography.. Biosci Rep.

[5] Manjunath P, Sairam MR (1987). Purification and biochemical characterization of three major acidic proteins (BSP-A1, BSP-A2 and BSP-A3) from bovine seminal plasma.. Biochem J.

[6] Chandonnet L, Roberts KD, Chapdelaine A, Manjunath P (1990). Identification of heparin-binding proteins in bovine seminal plasma.. Mol Reprod Dev.

[7] Esch FS, Ling NC, Bohlen P, Ying SY, Guillemin R (1983). Primary structure of PDC-109, a major protein constituent of bovine seminal plasma.. Biochem Biophys Res Commun.

[8] Seidah NG, Manjunath P, Rochemont J, Sairam MR, Cheretian M (1987). Complete amino acid sequence of BSP-A3 from bovine seminal plasma. Homology to PDC-109 and to the collagen-binding domain of fibronectin.. Biochem J.

[9] Desnoyers L, Manjunath P (1992). Major proteins of bovine seminal plasma exhibit novel interactions with phospholipids.. J Biol Chem.

[10] Thérien I, Moreau R, Manjunath P (1998). Major proteins of bovine seminal plasma and high-density lipoprotein induce cholesterol efflux from epididymal sperm.. Biol Reprod.

[11] Moreau R, Thérien I, Lazure C, Manjunath P (1998). Type II domains of BSP-A1/-A2 proteins: binding properties, lipid efflux and sperm capacitation potential.. Biochem Biophys Res Commun.

[12] Baker ME (1985). The PDC-109 protein from bovine seminal plasma is similar to the gelatin-binding domain of bovine fibronectin and a kringle domain of human tissue-type plasminogen activator.. Biochem Biophys Res Commun.

[13] Swamy MJ (2004). Interaction of bovine seminal plasma proteins with model membranes and sperm plasma membranes.. Curr Sci.

[14] Wah DA, Fernández-Tornero C, Sanz L, Romero A, Calvete JJ (2002). Sperm coating mechanism from the 1.8 Å crystal structure of PDC-109-phosphorylcholine complex.. Structure.

[15] Gasset M, Magdaleno L, Calvete JJ (2000). Biophysical study of the perturbation of model membrane structure caused by seminal plasma protein PDC-109.. Arch Biochem Biophys.

[16] Ramakrishnan M, Anbazhagan V, Pratap TV, Marsh D, Swamy MJ (2001). Membrane insertion and lipid-protein interactions of bovine seminal plasma protein, PDC-109 investigated by spin label electron spin resonance spectroscopy.. Biophys J.

[17] Greube A, Müller K, Töpfer-Petersen E, Herrmann A, Müller P (2001). Influence of the bovine seminal plasma protein PDC-109 on the physical state of membrane.. Biochemistry.

[18] Thomas CJ, Anbazhagan V, Ramakrishnan M, Sultan N, Surolia I (2003). Mechanism of membrane binding by the bovine seminal plasma protein, PDC-109. A surface plasmon resonance study.. Biophys J.

[19] Anbazhagan V, Swamy MJ (2005). Thermodynamics of phosphorylcholine and lysophosphatidylcholine binding to the major protein of bovine seminal plasma, PDC-109.. FEBS Lett.

[20] Lassiseraye D, Courtemanche L, Bergeron A, Manjunath P, Lafleur M (2008). Binding of bovine seminal plasma protein BSP-A1/A2 to model membranes: Lipid specificity and effect of the temperature.. Biochim Biophys Acta.

[21] Damai RS, Sankhala RS, Anbazhagan V, Swamy MJ (2010). ^31^P-NMR and AFM studies on the destabilization of cell and model membranes by the major bovine seminal plasma protein, PDC-109.. IUBMB Life.

[22] Manjunath P, Nauc V, Bergeron A, Menard M (2002). Major proteins of bovine seminal plasma bind to the low density lipoprotein fraction of hen's egg yolk.. Biol Reprod.

[23] Sankhala RS, Swamy MJ (2010). The major protein of bovine seminal plasma, PDC-109, is a molecular chaperone.. Biochemistry.

[24] Laskey RA, Honda BM, Mills AD, Finch JT (1978). Nucleosomes are assembled by an acidic protein which binds histones and transfers them to DNA.. Nature.

[25] Ellis RJ (1987). Proteins as molecular chaperones.. Nature.

[26] Gething MJ, Sambrook J (1992). Protein folding in the cell.. Nature.

[27] Horwitz J (1992). Alpha-crystallin can function as a molecular chaperone.. Proc Natl Acad Sci U S A.

[28] Hartl FU (1996). Molecular chaperones in cellular protein folding.. Nature.

[29] Rajaraman K, Raman B, Rao CM (1996). Molten-globule state of carbonic anhydrase binds to the chaperone-like alpha-crystallin.. J Biol Chem.

[30] Kumarasamy A, Abraham EC (2007). Interaction of C-terminal truncated human αA-Crystallins with target proteins.. PLoS ONE.

[31] Qu J, Susanne B-K, Holst O, Kleinschmidt JH (2009). Binding regions of outer membrane protein A in complexes with the periplasmic chaperone Skp. A site-directed fluorescence study.. Biochemistry.

[32] Das KP, Surewicz WK (1995). On the substrate specificity of α-crystallin as a molecular chaperone.. Biochem J.

[33] Datta SA, Rao CM (1999). Differential temperature-dependent chaperone-like activity of αA- and αB-crystallin homoaggregates.. J Biol Chem.

[34] Reddy GB, Das KP, Petrash JM, Surewicz WK (2000). Temperature-dependent chaperone activity and structural properties of human αA- and αB-crystallins.. J Biol Chem.

[35] Horwitz J, Huang Q, Ding L (2004). The native oligomeric organization of alpha-crystallin, is it necessary for its chaperone function?. Exp Eye Res.

[36] Sun Y, MacRae TH (2005). The small heat shock proteins and their role in human disease.. FEBS J.

[37] Guha S, Manna TK, Das KP, Bhattacharyya B (1998). Chaperone-like activity of tubulin.. J Biol Chem.

[38] Kim TD, Paik SR, Yang CH, Kim J (2000). Structural changes in α-synuclein affect its chaperone like activity in-vitro.. Protein Sci.

[39] Gasset M, Saiz JL, Sanz L, Gentzel M, Töpfer-Petersen E (1997). Conformational features and thermal stability of bovine seminal plasma protein PDC-109 oligomers and phosphorylcholine-bound complexes.. Eur J Biochem.

[40] Anbazhagan V, Damai RS, Paul A, Swamy MJ (2008). Interaction of the major protein from bovine seminal plasma, PDC-109 with phospholipid membranes and soluble ligands investigated by fluorescence approaches.. Biochim Biophys Acta.

[41] Damai RS, Anbazhagan V, Rao KB, Swamy MJ (2009). Fluorescence studies on the interaction of choline-binding domain B of the major bovine seminal plasma protein, PDC-109 with phospholipid membranes.. Biochim Biophys Acta.

[42] Calvete JJ, Paloma FV, Sanz L, Romero A (1996). A procedure for the large-scale isolation of major bovine seminal plasma proteins.. Protein Expr Purif.

[43] Lowry OH, Rosebrough NJ, Farr AL, Randall RJ (1951). Protein measurement with the Folin phenol reagent.. J Biol Chem.

[44] Hawe A, Rispens T, Herron JN, Jiskoot W (2010). Probing bis-ANS binding sites of different affinity on aggregated IgG by steady-state fluorescence, time-resolved fluorescence and isothermal titration calorimetry.. J Pharm Sci.

[45] Roberts DD, Goldstein IJ (1982). Hydrophobic binding properties of the lectin from lima beans (*Phaseolus lunatus*).. J Biol Chem.

[46] Kavitha M, Sultan NAM, Swamy MJ (2009). Fluorescence studies on the interaction of hydrophobic ligands with *Momordica charantia* (bitter gourd) seed lectin.. J Photochem Photobiol B: Biol.

[47] Liang JN, Li XY (1991). Interaction and aggregation of lens crystallins.. Exp Eye Res.

[48] Sharma KK, Kaur H, Kumar GS, Kester K (1998). Interaction of 1,1-bis(4-anilino)naphthalene-5,5-disulfonic acid with α-crystallin.. J Biol Chem.

[49] Kyte J, Doolittle RFJ (1982). A simple method for displaying the hydropathic character of a protein.. J Mol Biol.

[50] Anbazhagan V, Sankhala RS, Swamy MJ (2010). Thermodynamics of interaction of the major bovine seminal plasma protein, PDC-109 with phospholipid membranes.. An isothermal titration calorimetric study (Submitted for publication).

[51] Manček-Keber M, Jerala R (2006). Structural similarity between the hydrophobic fluorescent probe and lipid A as a ligand of MD-2.. FASEB J.

[52] Tannert A, Kurz A, Erlemann KR, Müller K, Herrmann A (2007). The bovine seminal plasma protein PDC-109 extracts phosphorylcholine-containing lipids from the outer membrane leaflet.. Eur Biophys J.

[53] Scolari S, Müller K, Bittman R, Herrmann A, Müller P (2010). Interaction of mammalian seminal plasma protein PDC-109 with cholesterol: implications for a putative CRAC domain.. Biochemistry.

[54] Bhattacharyya M, Ray S, Bhattacharya S, Chakrabarti A (2004). Chaperone activity and prodan binding at the self-associating domain of erythroid spectrin.. J Biol Chem.

[55] Welker S, Rudolph B, Frenzel E, Hagn F, Liebisch G (2010). Hsp12 is an intrinsically unstructured stress protein that folds upon membrane association and modulates membrane function.. Mol Cell.

