# Supplementary figures and images for: Correlation of Membrane Binding and Hydrophobicity to the Chaperone-Like Activity of PDC-109, the Major Protein of Bovine Seminal Plasma
Source: PLoS One. 2011 Mar 8;6(3):e17330. doi: 10.1371/journal.pone.0017330 (PMC3050878; doi:10.1371/journal.pone.0017330)

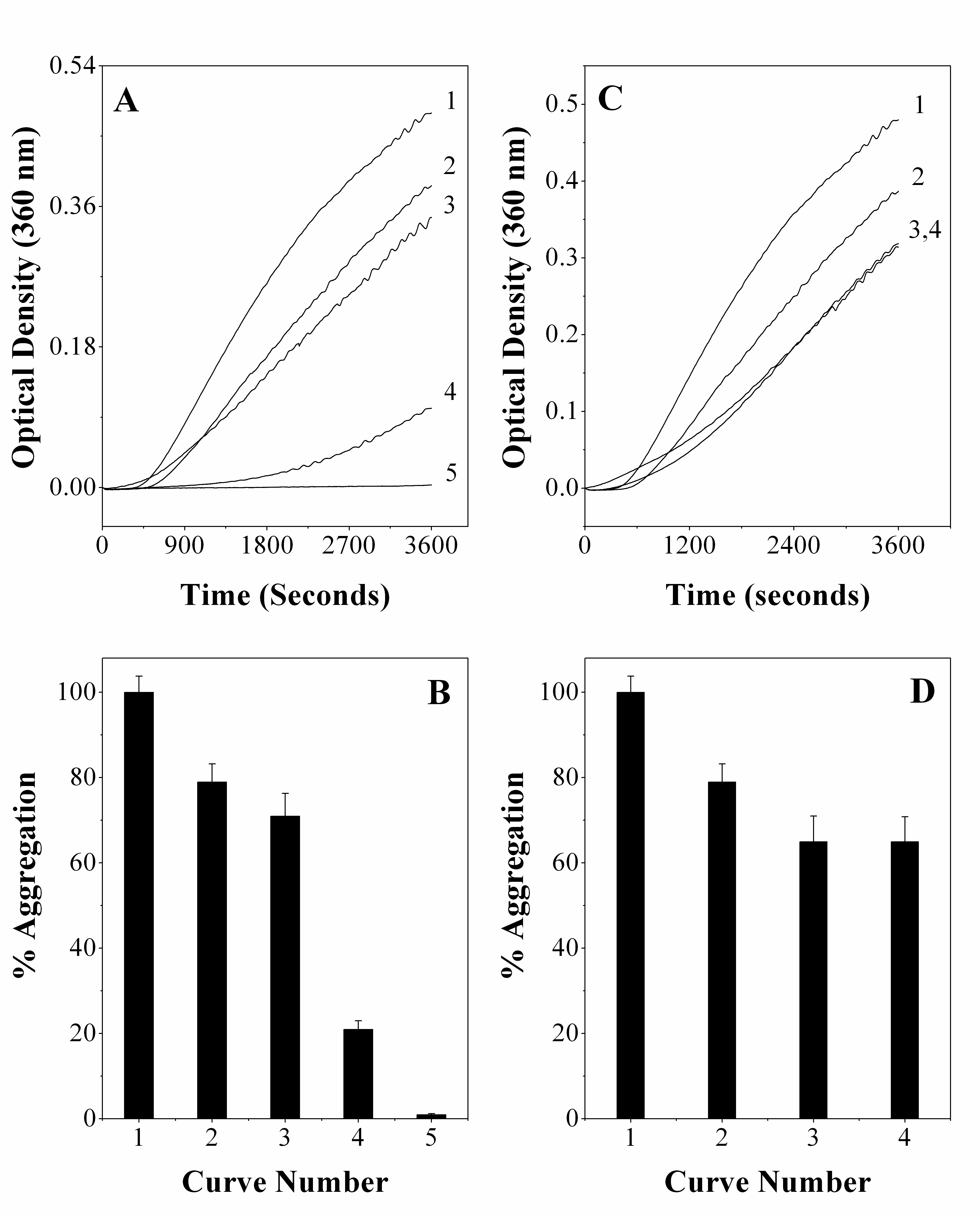

Supplement: Figure S1 — Effect of phospholipid binding on the CLA of PDC-109. A) Prevention of aggregation of CA (0.2 mg/ml) by PDC-109. Aggregation profiles of (1) Native CA at 52°C, (2) CA + 0.2 mg/ml PDC-109, (3) CA +5 µM of DMPC, (4) CA + PDC-109 (0.2 mg/ml) + DMPC (2 µM) and (5) CA + PDC-109 (0.2 mg/ml) + DMPC (5 µM) are shown. B) Bar diagram representing percent aggregation of CA under different conditions as shown in (A) at 3600 seconds. C) Aggregation profiles of (1) Native CA at 52°C, (2) CA + 0.2 mg/ml PDC-109, (3) CA + DMPG (2 µM) and (4) CA + PDC-109 (0.2 mg/ml) + DMPG (2 µM) are shown. D) Bar diagram for the data shown in (C) at 3600 seconds. (TIF) [file pone.0017330.s001.tif]

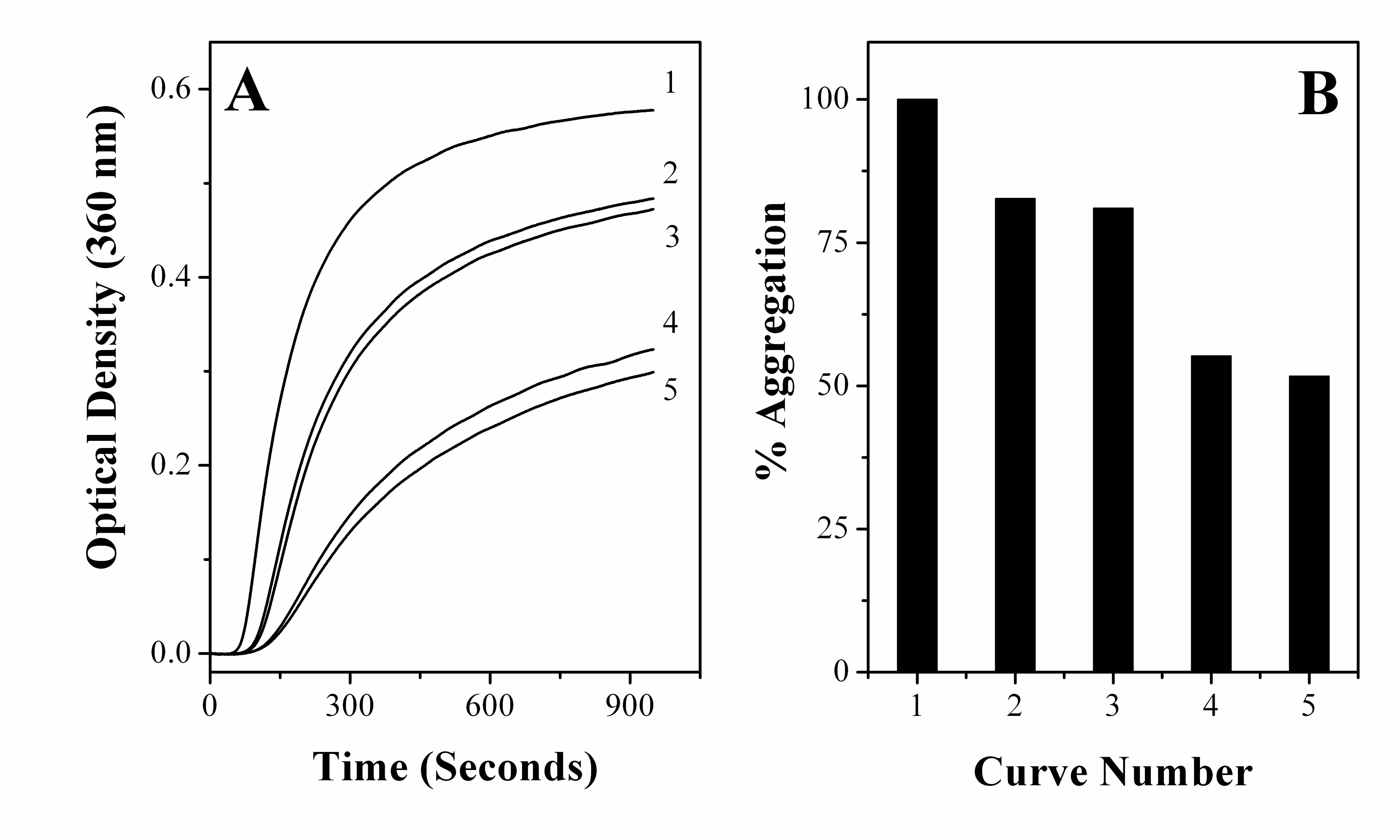

Supplement: Figure S2 — Effect of ANS binding on the CLA of PDC-109. Modulation of CLA of PDC-109 by ANS binding was investigated by aggregation assay with ADH (0.1 mg/mL) as the target protein. A) Aggregation profiles of (1) ADH at 48°C, (2) ADH + 0.025 mg/ml PDC-109, (3) ADH + 0.025 mg/ml ANS-PDC-109, (4) ADH + 0.05 mg/ml of PDC-109 and (5) ADH + 0.05 mg/ml of ANS-PDC-109 are shown. B) Bar diagram representing percent aggregation of LDH under different conditions as shown in (A) at 960 seconds. (TIF) [file pone.0017330.s002.tif]

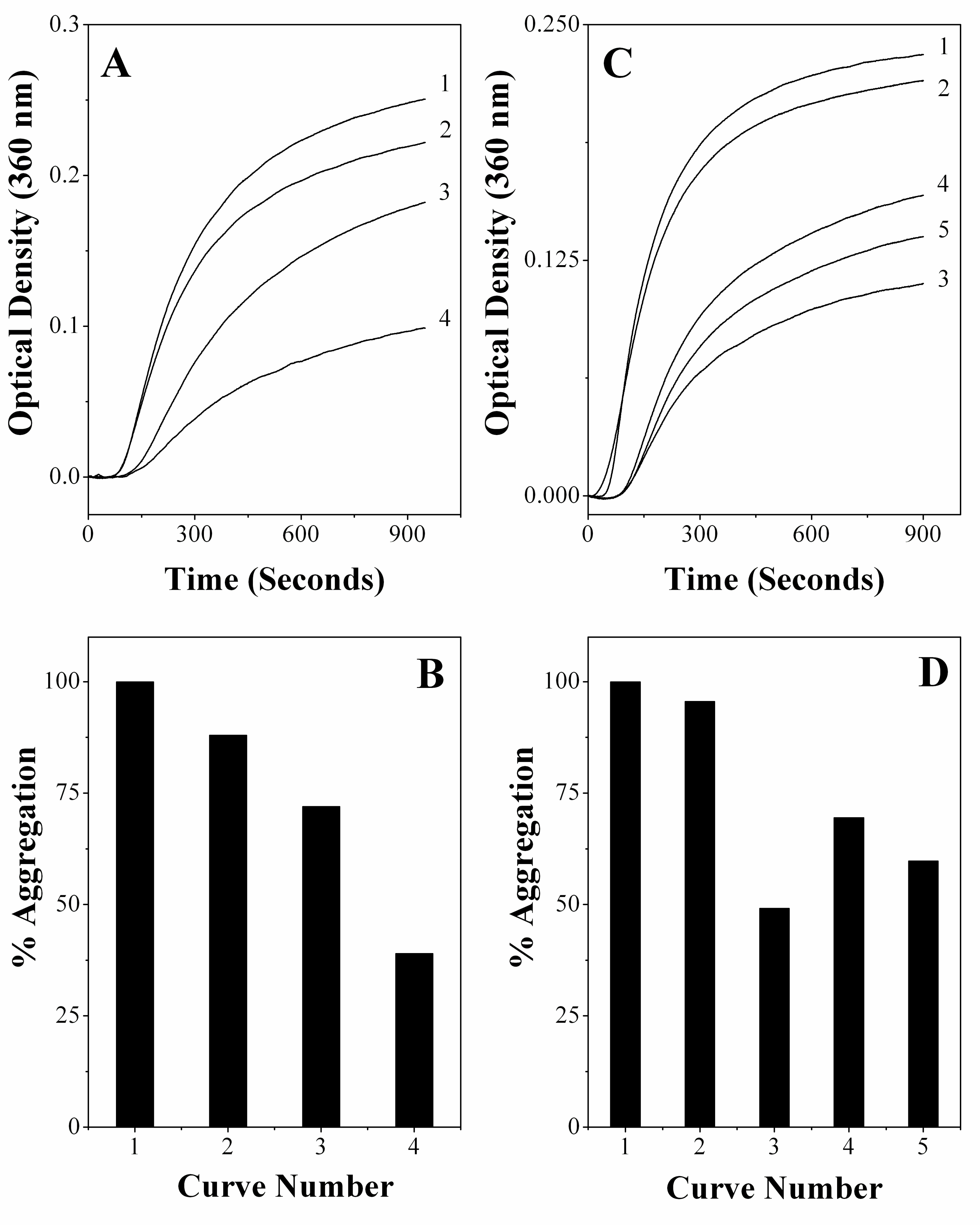

Supplement: Figure S3 — Effect of phospholipid binding on the CLA of PDC-109. Prevention of aggregation of ADH (0.05 mg/ml) by PDC-109. A) Aggregation profiles of (1) Native ADH at 48°C, (2) ADH + 2 µM of DMPC, (3) ADH + PDC-109 (0.025 mg/ml) and (4) ADH + PDC-109 (0.025 mg/ml) + DMPC (2 µM) are shown. B) Bar diagram representing percent aggregation of ADH under different conditions as shown in panel (A) at 960 seconds. C) Aggregation profiles of (1) Native ADH at 48°C, (2) ADH + DMPG (0.1 µM), (3) ADH + PDC-109 (0.03 mg/ml), (4) ADH + PDC-109 (0.03 mg/ml) + DMPG (0.05 µM) and (5) ADH + PDC-109 (0.03 mg/ml) + DMPG (0.1 µM) are shown. D) Bar diagram for the data shown in (C) at 900 seconds. (TIF) [file pone.0017330.s003.tif]

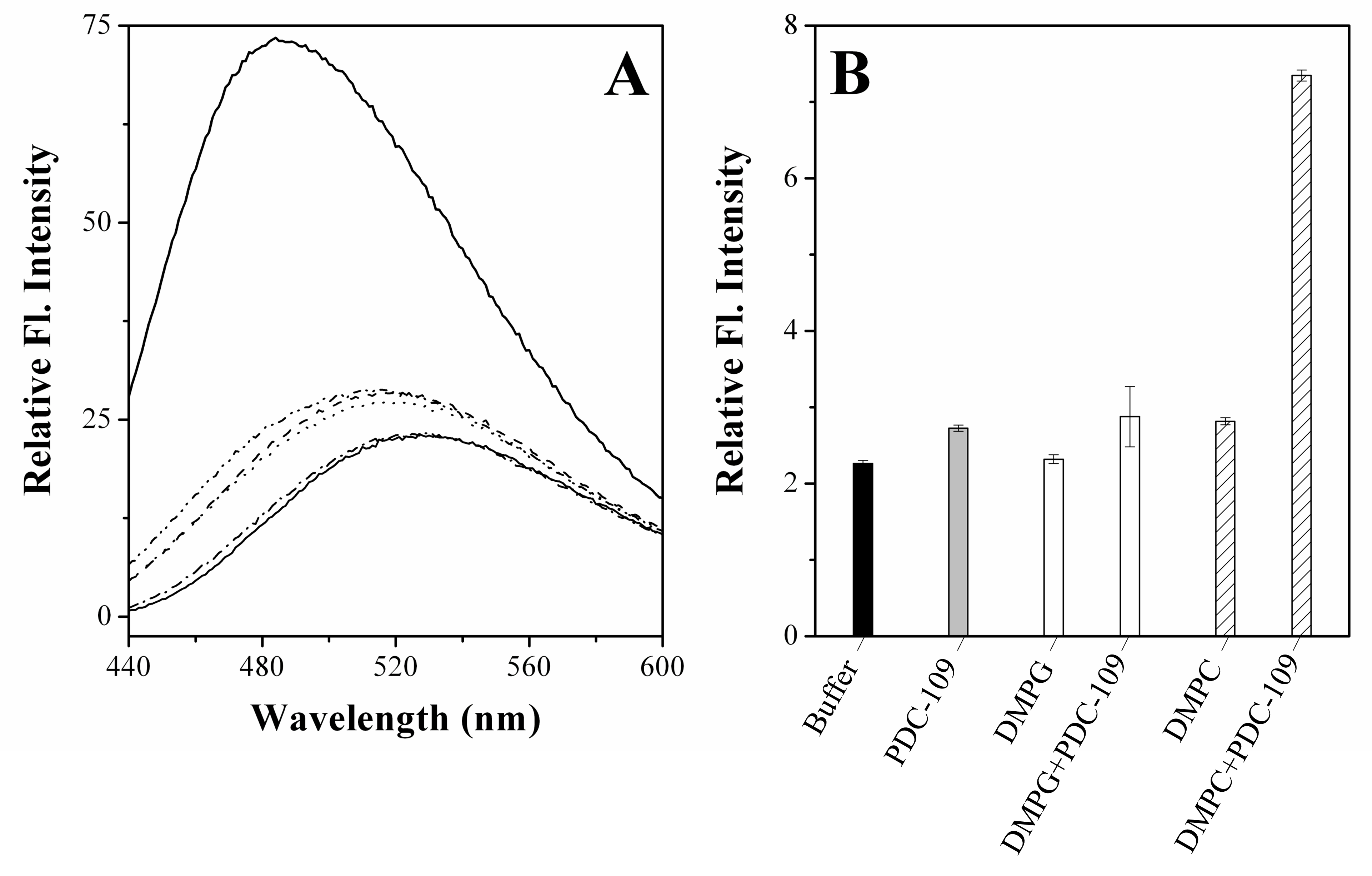

Supplement: Figure S4 — ANS Binding to phospholipids, PDC-109 and phospholipid-PDC-109 mixtures. A) Fluorescence spectra for the interaction of ANS with buffer (solid thin line), DMPG (5 µM, dash dot line), PDC-109 (0.05 mg/ml, dot line), DMPC (5 µM, dash line), DMPG-PDC-109 mixture (dash dot dot line) and DMPC-PDC-109 mixture (solid thick line) are shown. Final concentration of ANS in each sample was 50 µM. B) Relative fluorescence intensity of different samples at the emission maximum. (TIF) [file pone.0017330.s004.tif]

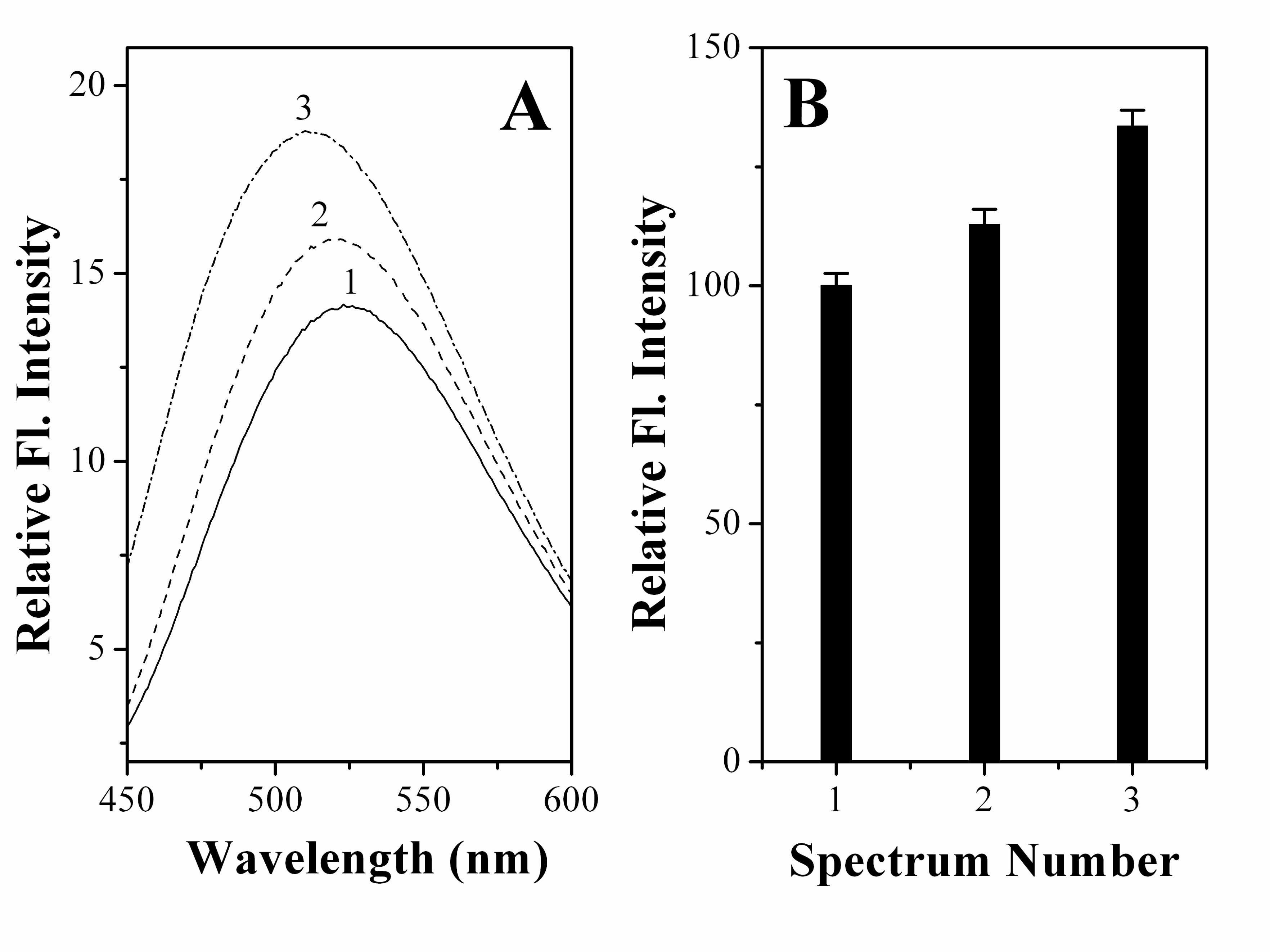

Supplement: Figure S5 — ANS Binding to PrC and PrC-PDC-109 mixtures. A) Fluorescence spectra of ANS in TBS-1 under different conditions. 1) with PrC; 2) with PDC-109 + PrC; 3) with PDC-109. Concentrations of different components used were: ANS, 50 µM; PDC-109, 0.05 mg/mL; PrC, 10 mM. B) Relative fluorescence intensity of different samples at the emission maximum. (TIF) [file pone.0017330.s005.tif]

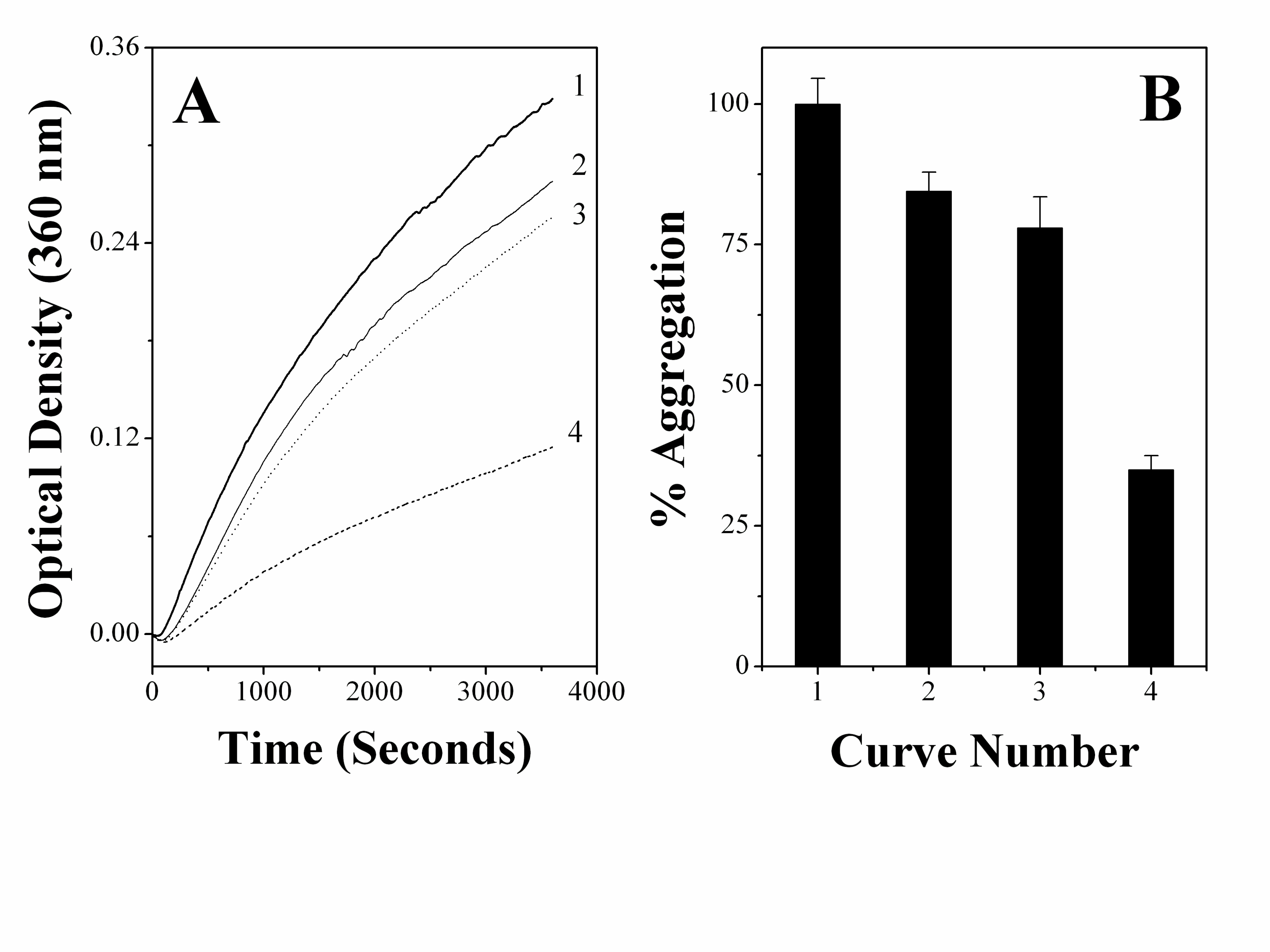

Supplement: Figure S6 — The Effect of cholesterol incorporation into phospholipid membrane, on the CLA of PDC-109. A) Prevention of aggregation of LDH (0.15 mg/ml). Aggregation profiles of (1) Native LDH at 50°C, (2) LDH + 0.075 mg/ml PDC-109, (3) LDH + PDC-109 (0.075 mg/ml) + DMPC/cholesterol (2 µM) and (4) LDH + PDC-109 (0.075 mg/ml) + DMPC (2 µM) are shown. B) Bar diagram representing percent aggregation of LDH under different conditions as shown in (A) at 3600 seconds. (TIF) [file pone.0017330.s006.tif]
