# Supplementary material for: Correlation of Membrane Binding and Hydrophobicity to the Chaperone-Like Activity of PDC-109, the Major Protein of Bovine Seminal Plasma
Source: PLoS One. 2011 Mar 8;6(3):e17330. doi: 10.1371/journal.pone.0017330 (PMC3050878; doi:10.1371/journal.pone.0017330)
Supplement: Text S1 — Methods and Results. Experimental details employed for studying the binding of ANS to PDC-109 and PDC-109/PrC complex and the results obtained from these experiments are provided. (DOC) [file pone.0017330.s007.doc]

**Correlation of Membrane Binding and Hydrophobicity to the Chaperone-Like Activity of PDC-109, the Major Protein of Bovine Seminal Plasma**

**Rajeshwer S. Sankhala, Rajani S. Damai and Musti J. Swamy***

*School of Chemistry, University of Hyderabad, Hyderabad-500046, Andhra Pradesh, India*

**Supporting Information**

**Methods**

**ANS binding to PDC-109 and PDC-109/phosphorylcholine mixtures.** Interaction of ANS with native PDC-109 and PDC-109/PrC mixtures was investigated at 25 ºC by steady-state fluorescence spectroscopy. ANS (50 µM) was added to the following solutions in TBS-I: 10 mM PrC, 0.05 mg/mL PDC-109, and 0.05 mg/mL PDC-109 containing 10 mM PrC. After incubation for 10 minutes, samples were excited at 350 nm and emission spectra were recorded between 450 and 600 nm using a Jobin-Yvon Spex Fluoromax 4 fluorescence spectrometer. Slit widths of 2 and 10 nm were used for the excitation and emission monochromators, respectively.

**Results**

**Modulation of surface hydrophobicity of PDC-109 by PrC binding.** ANS is a fluorescent probe that has been extensively used for identifying and characterizing hydrophobic regions in proteins and other biomolecules. Fluorescence spectra corresponding to ANS in buffer containing PrC and in the presence of PDC-109 and mixtures of PDC-109 with PrC are shown in Fig. S6A. Fluorescence intensity of ANS in buffer containing PrC (solid line) was considered as 100% and relative intensity of other samples was normalized with respect to it. Fluorescence intensity of ANS increased by ~33% in the presence of PDC-109 (dash dot line), whereas prior incubation of PDC-109 with PrC showed only 13% increase in the ANS fluorescence (dash line). A bar diagram indicating the relative fluorescence enhancement of various samples upon ANS binding is shown in Fig. S6B.

In our earlier study (Ref. 23, main manuscript) we have demonstrated that polydispersity is an important factor for the chaperone-like activity of PDC-109. The above results suggest that PrC binding decreases the surface hydrophobicity of the protein. It therefore appears that both dissociation of polydisperse aggregates and decrease in surface hydrophobicity, resulting from PrC binding have a negative impact on the CLA of PDC-109, indicating their importance for such an activity.
